# How green are large language models for radiology report labelling? Comparing human, rule-based and hybrid workflows

**DOI:** 10.1186/s13244-026-02289-2

**Published:** 2026-05-27

**Authors:** Matthias A. Fink, Arved Bischoff, Edem Atsiatorme, Alexander Kremer, Jonas Kroschke, Martin Moll, Patrick Stein, Veronika Riebl, Timo Leichenich, Hans-Ulrich Kauczor, Kai Schlamp

**Affiliations:** 1 Clinic for Diagnostic and Interventional Radiology, University Hospital Heidelberg, Heidelberg, Germany; 2 Translational Lung Research Center Heidelberg, Member of the German Center for Lung Research, Heidelberg, Germany; 3 Diagnostic and Interventional Radiology with Nuclear Medicine, Heidelberg Thoracic Clinic, University of Heidelberg, Heidelberg, Germany

**Keywords:** Structured reporting, Large language models, Data mining, Sustainability, Resource footprint

## Abstract

**Objectives:**

To address limited quantitative data on sustainable use of large language models (LLMs) in radiology, we quantified the resource footprint of LLMs for labelling CT pulmonary embolism reports and assessed how a hybrid rule-based–LLM workflow changes time, cost and carbon emissions compared with manual labelling.

**Materials and methods:**

In this single-centre retrospective study, 2923 structured CT reports were labelled using four workflows: a rule-based extractor (RBE), an LLM-only pipeline using 18 open-weight and four proprietary models, a hybrid RBE–LLM pipeline that routed RBE failures to an LLM, and full manual labelling by radiologists. Ground truth was based on radiologist adjudication. For each LLM, we measured per-report latency, estimated CO_2_ emissions and cost. Radiologists recorded the labelling time per report.

**Results:**

Manual labelling required 32.8 h for 2923 reports (40.4 s/report; €0.42/report) with 95.0% accuracy (95% CI: 93.7–96.2). LLM-only pipelines were less accurate (85.1%; 95% CI: 84.9–85.5) but reduced labelling time to 12.4 h and cost to €2.60 (both *p* < 0.001). Hybrid RBE–LLM workflows yielded the highest accuracy (98.5%) and lowest resource use: across 22 models, switching from LLM-only to hybrid reduced time (6.7 to 0.97 h), cost (€1.19 to €0.17), and CO_2_ (0.82 to 0.12 kg; all *p* < 0.001).

**Conclusion:**

LLM-only labelling reduced labour time and direct costs compared with manual annotation. A hybrid RBE–LLM pipeline that forwards rule-based failures to an LLM concentrated compute where needed and markedly decreased time, cost and emissions, supporting targeted deployment of LLMs for sustainable data-annotation workflows in radiology.

**Critical relevance:**

By quantifying time, cost and carbon emissions of manual, rule-based, LLM and hybrid report labelling, this study identifies sustainable workflows for deploying LLMs in routine radiology reporting.

**Key Points:**

- Manual expert labelling of CT pulmonary embolism reports is time-intensive and costly.
- Mid-sized LLM configurations provide favourable trade-offs between performance and resource use.
- Hybrid rule-based–LLM workflows sustain accuracy while reducing resource demands.

**Graphical Abstract:**

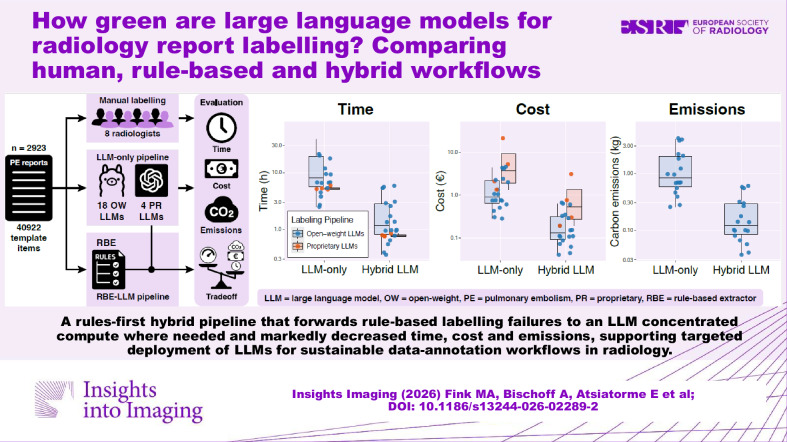

## Introduction

Artificial intelligence (AI) in radiology increasingly relies on programmatically generated labels rather than manual annotation. At scale, labels can be derived directly from routine reports using rule-based pipelines, weak supervision, and, more recently, large language models (LLMs), accelerating dataset curation while retaining clinically relevant information [1]. This practice underpins widely used resources such as MIMIC-CXR and CheXpert, where report-derived labels support downstream imaging research [2, 3]. Building on these datasets, the rule-based CheXpert labeller enabled automatic report labelling, and the transformer-based CheXbert subsequently improved label accuracy with a small set of expert annotations [3, 4].

However, the reliability and sustainability of programmatic labelling depend not only on model capability but also on how consistently reports are written and workflows are designed. Although professional societies actively promote structured reporting, such as RSNA’s RadReport templates and ESR position papers, routine adoption remains limited [5]. In many practices, template-based text is mixed with free-text dictation and later finalised by attending physicians after resident edits, introducing small but meaningful post hoc changes that challenge exact-match rules and motivate learning-based extraction [6–8].

Sustainability considerations further motivate workflow-oriented optimisation. Radiology departments have substantial baseline energy consumption, and a recent analysis reported that a large share of departmental energy use can be non-productive (e.g., scanners and workstations powered on but idle), with workflow interventions offering meaningful reductions in both energy use and costs [9]. In parallel, adoption is increasing: a 2024 survey among ESR members found that nearly half of respondents reported current AI use, and that LLM use is not limited to research, underscoring the relevance of understanding not only performance but also operational footprint [10].

Against this backdrop, LLMs can work with zero- or few-shot settings and may improve extraction robustness under heterogeneous phrasing [11–13]. Proprietary LLMs have shown strong performance, but concerns remain regarding data governance, reproducibility, and recurring access costs [14, 15]. Open-weight models (e.g., Llama and Qwen-family models) enable on-premises deployment and tighter privacy control [16–18]. However, inference on large models consumes significant energy and water, creating economic and environmental impacts that argue for targeted use [19, 20]. Recent lifecycle disclosures illustrate that the environmental burden of foundation models can be substantial, reinforcing the need to design workflows that concentrate compute where it adds value [21].

These considerations suggest a rules-first hybrid strategy: apply deterministic extraction for schema-conforming fields and invoke LLMs only for rule failures or ambiguous language. Structured CT pulmonary angiography (CTPA) reports for pulmonary embolism (PE) provide a practical test case because they contain enumerated fields yet remain heterogeneous across readers and clinical contexts [8].

The aim of this study was to quantify and compare the time, direct costs, and carbon emissions of radiology report labelling across four workflows—manual labelling, a rule-based extractor (RBE), a LLM-only pipeline, and a rules-first hybrid RBE–LLM pipeline—and to evaluate how workflow design enables resource-efficient, sustainable deployment of LLMs for report labelling in radiology.

## Materials and methods

### Study design and dataset

This retrospective single-centre benchmarking study was approved by the Ethics Committee of the University Hospital Heidelberg (S‑236/2020). Written informed consent was waived because only de-identified data were used. The structured PE template did not contain direct patient identifiers, and analyses were performed on report text and template fields stored under study-specific identifiers on secured institutional infrastructure. All CTPA examinations reported with a structured PE template between October 2021 and March 2025 were identified across a network of 14 hospitals within a shared radiology service. All CTPA examinations reported with the PE template during the study period were eligible; no additional exclusion criteria were applied. One report per patient was retained, yielding 2923 consecutive reports. The PE template contained 14 structured fields covering acquisition parameters, PE presence and burden, perfusion deficits, right-heart strain, image quality, and key impression items (Appendix E1). This dataset has been used in a companion diagnostic‑accuracy study that focuses on labelling performance of rule‑based, LLM-only and hybrid pipelines [22]; the present analysis concentrates on their resource footprint.

### Labelling workflows

**Fig. 1:**
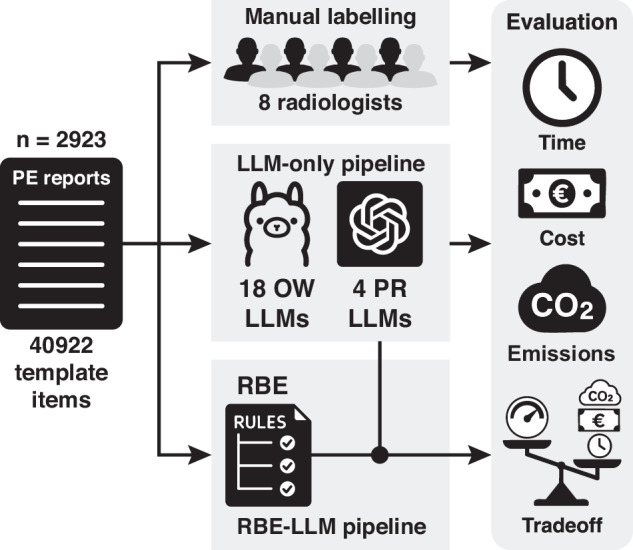
Study overview and labelling workflows. A total of 2923 CTPA reports documented with a structured PE template (14 fields; 40,922 labelled entries) were processed with four workflows: manual labelling by eight radiologists, a deterministic RBE, an LLM-only pipeline (18 open-weight and four proprietary LLMs), and a rules-first hybrid RBE–LLM pipeline. In the hybrid workflow, the RBE was applied first and only fields flagged as invalid (rule failures) were forwarded to the LLM; outputs were merged back on a per-field basis. For each workflow, cohort-level time, monetary cost, and (for open-weight models) estimated CO_2_ emissions were quantified under the study’s marginal execution boundary

Four workflows were compared, all populating the same 14‑field schema: an RBE, an LLM-only pipeline, a hybrid RBE–LLM pipeline and full manual labelling by radiologists. We use the term “LLM-only pipeline” for workflows in which the LLM populates all 14 schema fields for every report. We use a “hybrid RBE–LLM pipeline” for the rules-first design in which the RBE is applied first, and only fields flagged as invalid by the RBE (“RBE failures”) are forwarded to the LLM; LLM outputs are then merged back on a per-field basis. The schema (field definitions and allowed values) was specified a priori from the clinical template and was identical across all workflows (Appendix E1). Figure 1 summarises the study flow.

#### RBE

A deterministic rules engine mapped predefined textual patterns in the PE template to enumerated schema values. For each field, the RBE wrote a categorical code and flagged entries as missing (information not reported) or invalid (text incompatible with the schema, e.g. combined categories or free text).

#### LLM-only extractor

A model-agnostic LLM extractor queried 18 open-weight models and four generative pretrained transformer (GPT)-4 variants via a unified, structured-output interface (Appendix E2). A zero-shot prompt was used to describe the schema and request JavaScript Object Notation constrained by a fixed schema. Each model was processed once to generate a label vector containing 14 fields.

#### Hybrid RBE–LLM pipeline

In the hybrid RBE–LLM pipeline, the RBE was first applied to all reports. Fields labelled as valid by the RBE were retained, and only fields flagged as invalid (i.e., RBE failures) were sent to a selected LLM using the same structured-output interface. LLM outputs were merged back into the RBE table on a per-field basis, yielding paired LLM‑only and hybrid configurations for each of the 22 models.

#### Manual radiologist labelling

Eight radiologists (three board-certified radiologists and five residents with 0.7–9.6 years of experience) annotated the entire dataset using an electronic form mirroring the 14‑field schema. Each reader received a balanced, randomised assignment and was blinded to all automated outputs. An integrated timer recorded the labelling time per report.

### Reference standard and performance metrics

The RBE produced an initial label table for all reports. Two attending radiologists (M.A.F., 8 years of experience; K.S., 12 years of experience), blinded to all LLM outputs, adjudicated every field flagged as invalid by targeted review of the full report text. Where information could be mapped unambiguously to one of the allowed schema values, the corresponding code was entered; if information was missing, contradictory or ambiguous, the field remained invalid. Fields not flagged as invalid by the RBE were retained as extracted. This adjudicated RBE‑based table served as the reference standard.

### Measurement of time, CO_2_ emissions and costs

Primary resource endpoints were per-report and cohort-level inference time, energy use, CO_2_ emissions and monetary costs for each workflow. Resource estimates were computed from measured marginal execution during the experiments (wall-clock inference latency; for open-weight models, incremental workstation energy attributable to inference). They exclude model training and non-execution overheads (e.g., persistent infrastructure baseline, cooling, embodied hardware emissions), as well as development and maintenance effort for the rule-based and hybrid pipeline software. Accordingly, CO_2_ values should be interpreted as comparative, within-study estimates across workflows under a consistent boundary, not as a full lifecycle carbon footprint.

#### Inference time

For LLM-only workflows, inference latency was defined as wall‑clock time from sending the request to receipt of a schema‑valid response. Latencies were logged per report and summed to obtain cohort inference time; dividing by the number of reports yielded per‑report values. For hybrid configurations, measurements included the RBE pass and the subset of LLM calls triggered by RBE failures. For radiologists, per‑report labelling time was exported from the electronic form; mean time per report and projected cohort labelling time (mean × 2923) were computed per reader. For the RBE, wall-clock runtime for extracting the complete cohort (*n* = 2923 reports) was measured by rerunning the extractor 50 times within the same Python process after loading the dataset once and is reported as mean ± standard deviation across runs.

#### Energy use and CO_2_ emissions

Open-weight models were served locally via Ollama on a workstation equipped with an NVIDIA Quadro RTX 8000 (48 GB VRAM), a 12-core Intel Xeon central processing unit (CPU), and 128 GB RAM. Energy during inference was measured on this workstation using pyJoules with CPU/graphics processing unit (GPU) energy counters. All open-weight models were served through the same local Ollama setup and queried via an identical structured-output interface and prompt. Each LLM call was wrapped in a measurement context, and incremental energy was summed across reports, converted to kilowatt-hours (kWh), divided by the number of reports to obtain per‑report values and multiplied by 2923 for cohort totals. CO_2_ emissions were estimated by multiplying incremental energy by the German grid emission factor (kg CO_2_ per kWh) published by the Federal Environment Agency for the study period [23]. For proprietary GPT‑4 variants, provider‑level energy or emission data were not available, so only latency and monetary costs are reported.

#### Monetary costs

For open-weight LLMs, incremental energy consumption was multiplied by the commercial electricity price (€/kWh) of the academic centre to estimate per‑report and cohort electricity costs. For proprietary models, costs were derived from token‑based charges according to the provider’s public pricing at the time of the experiments and normalised per report and per cohort. For the radiologist baseline, tariff-based gross wages were converted into hourly rates and multiplied by the recorded labelling time per report to obtain labour costs.

### Statistical analysis

Analyses were performed by one author (M.A.F.) using R (version 4.5.1) and Python (version 3.13). Continuous variables are reported as means with 95% confidence intervals or medians with interquartile ranges (IQRs), as appropriate. Where applicable, 95% confidence intervals were estimated using non‑parametric bootstrapping with 1000 resamples. Paired comparisons of LLM-only vs hybrid pipelines used the Wilcoxon signed-rank test; comparisons of open‑weight vs proprietary models used the Mann–Whitney *U*-test. Pareto-front analyses related item‑level F1 scores to time, cost and emissions to identify models with favourable performance–resource trade-offs. A *p*-value < 0.05 was considered statistically significant.

## Results

### Baseline radiologist workload and variability

We analysed CTPA reports from 2923 unique patients (mean age, 66 ± 17 years; 50.1% female). All examinations were documented using a structured PE template comprising 14 structured fields, yielding 40922 labelled entries. Manual labelling of the entire cohort by eight radiologists took 32.8 h (range, 23.7–38.7 h) and incurred a direct labour cost of €1229.50 (range, €773.50–€1688.70) for 2923 reports (Table 1). This equates to a median of 40.4 s (range, 16–150 s) and 42.1 cents (range, 26.5–57.8 cents) per report (Appendices E3 and E4).

**Table 1:** Accuracy and resource use of labelling pipelines

Parameter	Accuracy (%)^*^	Cohort time (h)	Cohort cost (€)	Cohort CO_2_ (kg)
Radiologists	95.0 (93.7, 96.2)	32.8 (23.7, 38.7)	1229.50 (773.50, 1688.70)	NA
RBE	91.3 (90.7, 92.6)	< 0.001^‡^	NA^†^	NA^†^
LLM-only pipeline	85.1 (84.9, 85.5)	12.4 (2.5, 40.7)	2.60 (0.30, 21.10)	1.4 (0.3, 4.1)
Open-weight LLM	84.1 (83.6, 84.4)	14.0 (2.5, 40.7)	1.60 (0.30, 4.50)	1.4 (0.3, 4.1)
Proprietary LLM	89.8 (88.8, 90.3)	5.4 (5.0, 5.9)	7.40 (1.30, 21.10)	NA
*p-*value	0.34	0.12	0.03	
Hybrid LLM pipeline	98.5 (98.3, 98.6)	1.8 (0.4, 5.9)	0.40 (0, 3.10)	0.2 (0.04, 0.6)
Open-weight LLM	98.4 (98.2, 98.5)	2.0 (0.4, 5.9)	0.2 (0, 0.70)	0.2 (0.04, 0.6)
Proprietary LLM	98.9 (98.9, 99.1)	0.8 (0.8, 0.9)	1.10 (0.20, 3.10)	NA
*p-*value	0.14	0.14	0.03	

Unless otherwise specified, data are means with ranges in parentheses. Values represent mean cohort-level resource use per pipeline family; *p*-values compare open-weight vs proprietary LLMs within each pipeline type. Radiologists cohort time and cost were projected from each reader’s mean per-report time and hourly gross wage, and summarised as mean with range across eight readers

*NA* not available, *LLM* large language model

^*^ Denotes report-level accuracy, defined as all template fields of a report matching the reference standard and is shown with 95% confidence intervals

^†^ Monetary cost and CO_2_ emissions were not metered for the RBE

^‡^ RBE cohort runtime was measured by rerunning the extractor 50 times on the full cohort

### Pipeline-level resource use: radiologists vs LLM-only workflows

Table 1 summarises report-level accuracy and cohort-level resource use across pipeline families. Report-level accuracy was defined as all 14 fields matching the adjudicated reference standard for a given report. The RBE served as a deterministic baseline and achieved 91.3% report-level accuracy (95% CI: 90.7–92.6) (Table 1). In a dedicated runtime benchmark (50 repeated runs on the full cohort), the RBE processed all 2923 reports in 0.767 ± 0.004 s, corresponding to < 0.001 h of cohort processing time and approximately 0.26 ms per report. LLM-only pipelines had a report-level accuracy of 85.1% (95% CI: 84.9–85.5) compared with 95.0% (95% CI: 93.7–96.2) for radiologists. At this accuracy level, mean cohort labelling time decreased from 32.8 h for radiologists to 12.4 h for LLM-only pipelines (*p* < 0.001), and projected cohort cost from €1229.50 to €2.60 (*p* < 0.001). For open-weight LLM-only configurations, the corresponding estimated cohort CO_2_ footprint was 1.4 kg; CO_2_ estimates were not available for proprietary models or for radiologists. Within the LLM-only configurations, open-weight models showed a mean report-level accuracy of 84.1% (95% CI: 83.8–84.6) compared with 89.8% for proprietary models (95% CI: 89.0–90.2; *p* = 0.34). Mean cohort time was 14.0 h for open-weight and 5.4 h for proprietary pipelines (*p* = 0.12), while mean cohort cost was €1.60 and €7.40, respectively (*p* = 0.03). Hybrid RBE–LLM pipelines had the highest overall accuracy, with a mean report-level accuracy of 98.5%. For open-weight hybrids, the mean cohort labelling time was 1.8 h, the cohort cost €0.40, and the estimated cohort CO_2_ footprint was 0.2 kg. Within the hybrid family, open-weight and proprietary models had similar mean accuracy (98.4% vs 98.9%; *p* = 0.16) and cohort time (2.0 h vs 0.8 h; *p* = 0.14), whereas cohort cost differed (€0.20 vs €1.10; *p* = 0.03).

### Within-model impact of hybridisation

**Fig. 2:**
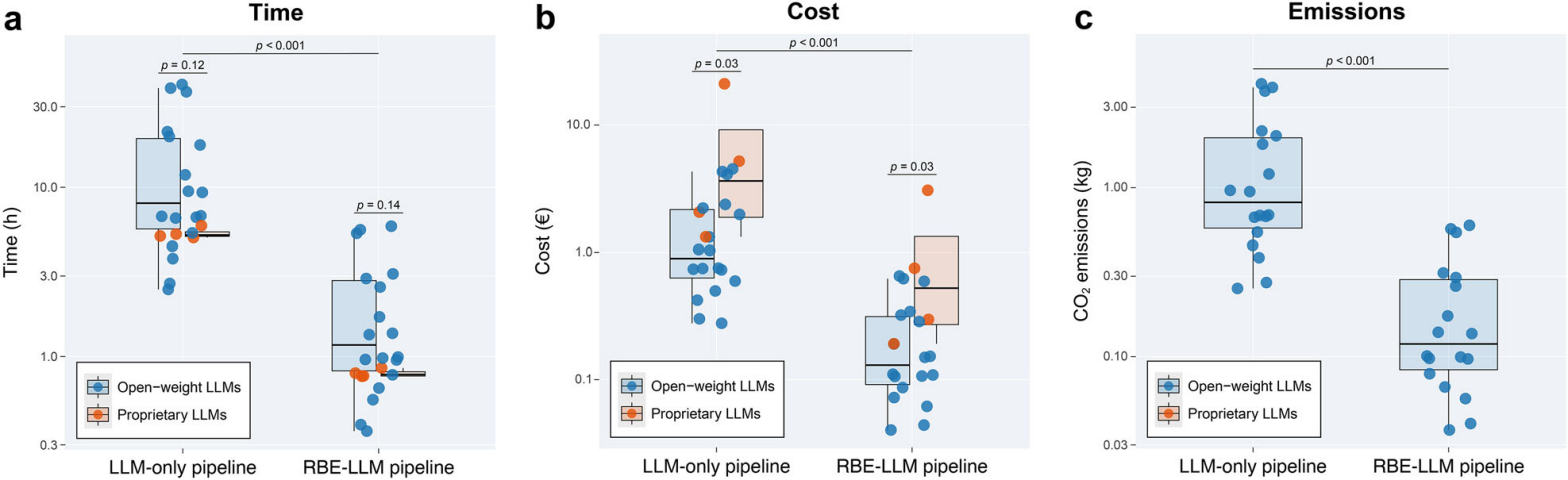
Cohort-level resource footprint of LLM workflows. Cohort-level (*n* = 2923 reports) (**a**) processing time, (**b**) monetary cost, and (**c**) estimated CO_2_ emissions are shown for LLM-only and hybrid RBE–LLM pipelines across 22 LLM configurations. Points represent individual models; boxes depict the median and IQR, and whiskers the 5th–95th percentiles. Axes are shown on a logarithmic scale. Horizontal brackets report two-sided Wilcoxon signed-rank *p*-values for paired comparisons (LLM-only vs hybrid for the same model) and two-sided Mann–Whitney *U*-test, *p*-values for unpaired comparisons (open-weight vs proprietary models within the same pipeline). CO_2_ emissions are shown for open-weight models only, as provider-level emission data were not available for proprietary models. The RBE baseline is not shown because its cohort runtime (0.767 ± 0.004 s) is orders of magnitude smaller than LLM inference on the logarithmic scale

To separate the impact of pipeline design from that of model selection, Table 2 and Fig. 2 provide paired comparisons of LLM-only and hybrid RBE–LLM configurations for the same 22 LLMs. Switching from LLM-only to hybrid processing reduced the median cohort labelling time across models from 6.7 h (IQR: 5.2–16.3 h) to 1.0 h (IQR: 0.8–2.4 h), corresponding to a median paired reduction of 5.7 h (*p* < 0.001). Median cohort cost decreased from €1.19 (IQR: €0.73–€2.34) with LLM-only workflows to €0.17 (IQR: €0.11–€0.34) in the hybrid setting: a median reduction of €1.01 per model run (*p* < 0.001). A similar pattern was observed for open-weight models (Table 2 and Fig. 2), with median emissions decreasing from 0.82 to 0.12 kg (*p* < 0.001).

**Table 2:** Cohort-level resource use with hybrid vs LLM-only pipelines

Parameter	*n*	LLM-only pipeline	Hybrid LLM pipeline	Difference (hybrid-LLM-only)	*p*-value
Time (h, cohort)	22	6.7 (5.2, 16.3)	1.0 (0.8, 2.4)	−5.7	< 0.001
Cost (€, cohort)	22	1.19 (0.73, 2.34)	0.17 (0.11, 0.34)	−1.01	< 0.001
CO_2_ emissions (kg, cohort)^†^	18	0.82 (0.58, 1.98)	0.12 (0.08, 0.29)	−0.70	< 0.001

Data are medians with IQRs in parentheses, summarising paired comparisons across 22 LLM configurations (LLM-only vs hybrid LLM pipelines). Difference denotes the median paired difference (hybrid-LLM-only). *p*-values are derived from Wilcoxon signed-rank tests for paired samples (*n* = 22 for labelling time and cost; *n* = 18 for CO_2_ emissions)

^†^ Only open-weight models; emissions for proprietary models were not available

### Performance–resource trade-offs of LLM pipelines

**Fig. 3:**
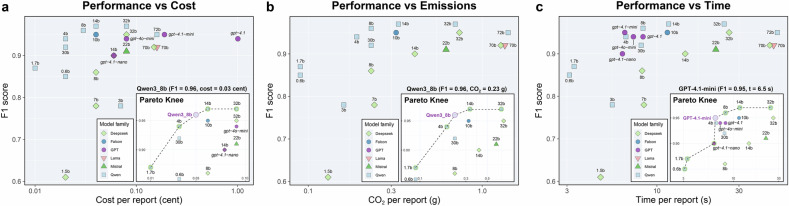
Performance–resource trade-off and Pareto analysis for LLM-only workflows. Item-level F1 scores are plotted against per-report (**a**) cost, (**b**) CO_2_ emissions, and (**c**) time for all LLM-only configurations. Marker shape indicates model family, and point labels denote model size/variant. X-axes are shown on a logarithmic scale to accommodate the observed order-of-magnitude variation. Insets highlight Pareto-efficient configurations and the corresponding “Pareto knee” (best-balanced trade-off in each panel). CO_2_ estimates are available for open-weight models only; proprietary models are therefore not shown in the emissions panel

Figure 3 shows the performance–resource landscape for all LLM-only workflows. For similar item-level F1 scores, per-report time, cost and CO_2_ emissions varied by more than an order of magnitude, and a subset of open-weight and proprietary models formed a Pareto front combining high F1 with low resource use (Table 3). Among open-weight models, Qwen3-8b, Qwen3-14b and Qwen3-32b lay near the Pareto knee, with item-level F1 scores of 0.96–0.97, report-level accuracies of 92.6–95.5% and per-report times of 8.3–24.6 s. Their costs ranged from 0.03 to 0.08 cents per report and estimated CO_2_ emissions from 0.23 to 0.70 g per report. Compared with the pooled radiologist baseline, these Pareto-efficient open-weight configurations reduced mean time per report by 39–79% and direct monetary cost by 99.8–99.9% (Table 3 and Appendix E3). Smaller models such as Qwen3-1.7b and Qwen3-0.6b further reduced resource use to 3.1–3.3 s, 0.01 cent and approximately 0.09 g CO_2_ per report, at lower report-level accuracies of 54.3–78.6%. Among proprietary models, GPT-4.1-mini was Pareto-efficient, with an item-level F1 of 0.98, report-level accuracy of 96.1% and 6.5 s per report, but a higher token-based cost of 0.18 cent per report and no available CO_2_ estimates from provider documentation.

**Table 3:** Performance and per-report resource use for Pareto-efficient LLM workflows

Parameter	F1 score	Accuracy (%)	Time per report (s)	Cost per report (cent)	CO_2_ per report (g)^*^	Time vs radiologists (% change)	Cost vs radiologists (% change)
Qwen3-32b	0.97 (0.96, 0.97)	94.1 (93.1, 94.8)	24.6 (21.4, 32.1)	0.08 (0.07, 0.10)	0.70 (0.60, 0.91)	−39.0%	−99.8%
Qwen3-14b	0.96 (0.96, 0.96)	95.5 (94.6, 96.3)	11.9 (10.3, 15.9)	0.04 (0.03, 0.05)	0.33 (0.29, 0.45)	−71.1%	−99.9%
Qwen3-8b	0.96 (0.96, 0.96)	92.6 (91.7, 93.8)	8.3 (6.2, 11.4)	0.03 (0.02, 0.04)	0.23 (0.17, 0.32)	−79.4%	−99.9%
Qwen3-1.7b	0.87 (0.86, 0.88)	78.6 (76.9, 79.5)	3.3 (2.7, 5.2)	0.01 (0.01, 0.02)	0.09 (0.08, 0.15)	−91.7%	−100.0%
Qwen3-0.6b	0.86 (0.85, 0.86)	54.3 (52.5, 56.6)	3.1 (0, 6.4)	0.01 (0, 0.02)	0.09 (0.07, 0.18)	−92.4%	−100.0%
GPT-4.1-mini	0.98 (0.97, 0.98)	96.1 (95.3, 96.8)	6.5 (3.4, 40)	0.18 (0.16, 0.21)	NA	−83.9%	−99.6%

Values summarise item-level F1 and report-level accuracy with 95% confidence intervals, and per-report resource use for LLM-only workflows near the Pareto knee of the performance–resource tradeoff. Time and cost vs radiologists indicate the percentage change in mean per-report time and cost relative to the pooled radiologist baseline

^*^ CO_2_ estimates are only available for open-weight models

## Discussion

This resource-focused analysis of 2923 structured CTPA reports quantified the time, monetary cost, and CO_2_ emissions associated with manual, LLM-only, and hybrid RBE–LLM labelling workflows. Although manual annotation by eight radiologists provided a high-quality human reference, it required 32.8 h of projected cohort time and approximately €1230 in labour costs and achieved 95.0% report-level accuracy (95% CI: 93.7–96.2). LLM-only pipelines were less accurate (85.1%; 95% CI: 84.9–85.5) but completed labelling in a much shorter time and at a much lower cost, while hybrid RBE–LLM pipelines achieved the highest level of accuracy (98.5%) and the lowest level of resource use. Switching from an LLM-only to a hybrid workflow for the same set of 22 models reduced median cohort labelling time from 6.7 to 0.97 h, cost from €1.19 to €0.17, and CO_2_ emissions from 0.82 to 0.12 kg. These findings emphasise the importance of workflow design in the sustainable deployment of LLMs. This workflow-centric perspective is consistent with institutional and society-level sustainability efforts in radiology, such as the ESR “Green Imaging Department (Green ID)” initiative, which promotes operational monitoring and optimisation at the department level [24].

Rule-based systems such as CheXpert, as well as transformer-based successors like CheXbert, have demonstrated that weakly supervised labels can support the development of high-quality downstream models [4]. Meanwhile, recent GPT-based approaches have shown that LLMs can match or even outperform these labellers when it comes to chest radiography reports [25, 26]. In this context, our study provides prospective, task-level measurements of time, cost, and emissions for a multi-model LLM benchmark in CT PE reporting. Within this structured-template setting, hybrid RBE–LLM pipelines reached and exceeded radiologist report-level accuracy, whereas LLM-only extraction remained below the radiologist baseline. This indicates that the main gains in both performance and sustainability derive from workflow design (rules-first with targeted LLM salvage), rather than from applying LLMs to all reports.

The advantages of hybrid RBE–LLM pipelines are consistent with findings from other fields where deterministic rules are combined with LLMs [27, 28]. Previous research has demonstrated that applying rules initially and only invoking LLMs for residual uncertainty can outperform both rule-based and LLM-only approaches [29]. Our results generalise this principle across 22 models and quantify the associated savings: routing only RBE-invalid reports to an LLM preserved or improved accuracy while substantially reducing time, cost, and emissions. This supports a “rules-first, LLM-second” approach wherever the structure of the report allows, reserving LLM capacity for genuinely ambiguous or off-template content. Recent work similarly indicates that well-specified annotation guidelines and structured extraction rules can improve LLM-only information extraction, conceptually supporting rules-first hybrid designs. More broadly, this aligns with a “machines-in-the-loop” paradigm, in which deterministic components and targeted AI assistance are combined to achieve reliable, auditable workflows [30, 31].

From a sustainability perspective, these data highlight the emerging “AI sustainability paradox” in radiology, whereby performance improvements could be counterbalanced by environmental and economic costs if resource usage is not actively managed [32]. Recent lifecycle assessments of large models, such as Mistral Large-2, have documented significant emissions and water usage during training and inference, and broader analyses have revealed substantial environmental impacts for routine LLM inference [20, 21, 33]. In our study, the absolute emissions per report for open-weight models were modest (in the low-gram range), but the relative differences between workflows were significant. This suggests that the cumulative institutional impact will depend less on choosing a single “best” model, and more on whether LLM calls are tightly controlled within hybrid pipelines.

When comparing open-weight and proprietary models, data governance, cost and infrastructure constraints in healthcare must also be considered [34]. Because provider-level emissions are not disclosed for proprietary application programming interfaces (APIs), direct environmental comparisons between open-weight and proprietary deployments are not possible in this study; CO₂ results are therefore reported for open-weight models only. Cloud-hosted proprietary LLMs raise concerns regarding privacy, regulatory compliance, and recurring API charges, factors that could hinder their adoption, particularly in systems with limited resources [35, 36]. Open-weight models allow for on-premises deployment, providing local control over data flows and electricity tariffs [37–39]. In our experiment, mid-sized open-weight models close to the Pareto front achieved a balance between high accuracy and short runtimes, very low incremental cost, and comparatively low CO_2_ emissions. However, on-premises deployments may incur a system-level energy baseline that is not captured by per-call inference measurements (e.g., continuous server uptime, GPU/CPU idling, memory allocation, cooling, and redundancy). Similarly, token-based fees represent marginal execution costs and may change over time; implementation decisions should therefore distinguish marginal inference costs from the total cost of ownership (hardware, maintenance, orchestration, and staffing). These considerations reinforce the value of hybrid routing policies that minimise the number of LLM calls in routine operations. Beyond structured templates, LLMs have also been used to transform free-text reports into structured, score-based outputs, suggesting that structured-output approaches may extend to less templated reporting, although resource footprints and failure modes will remain task- and site-dependent [40].

These results have several practical implications for radiology services. For example, replacing the repeated manual labelling of structured reports with hybrid RBE–LLM pipelines could free up several days’ worth of radiologist time for every few thousand examinations. This would enable expert effort to be reallocated to activities that clearly require human judgement, such as protocol optimisation, multidisciplinary case conferences and patient communication. Quantified time, cost and CO_2_ metrics could inform decisions regarding the procurement and architecture of open-weight models vs reliance on proprietary cloud services. Furthermore, the straightforward measurement routines employed here—code-level energy metering and per-pipeline accounting of time and cost—demonstrate how sustainability metrics could be incorporated into institutional dashboards for AI tools. In practice, sustainability-oriented implementation could include batch processing during scheduled windows, utilisation monitoring to reduce idle power, and strict routing policies that forward only low-confidence or out-of-schema cases to LLM inference.

This study has limitations. It is based on structured CTPA reports from one regional network using a single PE template, which may limit generalisability to other modalities, organ systems, or fully free-text reporting. The reference standard was derived from a rules-first table: only fields flagged as invalid by the RBE underwent targeted radiologist adjudication, whereas RBE-valid fields were retained. Therefore, systematic RBE errors in fields not flagged as invalid could remain undetected and may bias comparisons in favour of RBE-containing workflows. This risk may be lower in structured templates with enumerated value sets, but it cannot be excluded. Resource estimates reflect marginal execution under our study test conditions. Radiologist labour costs reflect compensated expert clinical judgement and responsibility, whereas LLM costs represent inference-level execution only under the marginal execution boundary. Thus, cost comparisons should be interpreted within this marginal boundary rather than as a substitution statement. For open-weight models, emissions were derived from incremental inference-time electricity on the measured workstation and a national grid factor. Embodied hardware emissions, cooling, and infrastructure overheads were not included, and real-world deployments may entail additional baseline energy use and “hidden” costs (e.g., server uptime, GPU/CPU idling, orchestration/monitoring, software updates, and IT staffing) relevant to the total cost of ownership. We did not quantify the up-front development and ongoing maintenance effort required to create and sustain the RBE and hybrid pipeline (e.g., rule engineering, validation, monitoring, and iterative updates), which is typically amortised over repeated cohorts and institutional reuse; therefore, its per-report impact decreases with scale. Emissions and hardware-related costs for proprietary GPT-4 variants could not be assessed due to missing provider data; proprietary results are therefore reported for latency and token-based fees only. Finally, our analysis focused on labelling accuracy and resource footprints; the impact of residual labelling errors on specific clinical or research outcomes requires task-specific evaluation.

In conclusion, rules-first hybrid RBE–LLM pipelines can match or exceed radiologist report-level accuracy while reducing time, monetary cost, and CO_2_ emissions for labelling structured CT reports. Once a clinically acceptable performance threshold has been reached, optimising pipeline design and routinely measuring resource use seem essential to align LLM deployment for radiology data labelling with environmental and economic sustainability goals.

## Supplementary information

ELECTRONIC SUPPLEMENTARY MATERIAL

## Data Availability

The code for the rule-based and LLM labelling pipelines, together with configuration files and example schemas, is available in a public GitHub repository. Due to data protection regulations and institutional policies, the full set of original clinical reports cannot be shared publicly. Access to pseudonymised label tables may be considered on reasonable request and subject to data-sharing agreements with the University Hospital Heidelberg.

## Abbreviations

AI: Artificial intelligence
API: Application programming interface
CPU: Central processing unit
CTPA: CT pulmonary angiography
GPT: Generative pretrained transformer
GPU: Graphics processing unit
IQR: Interquartile range
LLM: Large language model
PE: Pulmonary embolism
RBE: Rule-based extractor
