# Supplementary material for: How green are large language models for radiology report labelling? Comparing human, rule-based and hybrid workflows
Source: Insights Imaging. 2026 May 27;17:142. doi: 10.1186/s13244-026-02289-2 (PMC13216412; doi:10.1186/s13244-026-02289-2)
Supplement: Supplementary file 1 — ELECTRONIC SUPPLEMENTARY MATERIAL [file 13244_2026_2289_MOESM1_ESM.docx]

**How green are large language models for radiology report labelling?**

**Comparing human, rule-based and hybrid workflows**

**ELECTRONIC SUPPLEMENTARY MATERIAL**

## Appendix E1: Label schema: extracted fields and allowed values

| **Template item** | **Section** | **Data type** | **Allowed values** |
| --- | --- | --- | --- |
| ECG synchronization | Scan parameters | Categorical | ECG sync; no ECG sync |
| Attenuation of main pulmonary artery | Scan parameters | Numeric | Number in HU (e.g., 484) |
| Artifact score | Scan parameters | Ordinal | 0; 1; 2; 3; 4; 5 |
| Evidence of pulmonary embolism | PE findings | Categorical | Presence of PE; no presence of PE; suspicion of PE; not assessable |
| Clot burden score | PE findings | Numeric | Decimal number (e.g. 15.5) |
| Perfusion deficit (DE-CT) | PE findings | Categorical | None; < 25%; ≥ 25% |
| RV/LV ratio category | PE findings | Categorical | RV/LV ratio < 1; RV/LV ratio ≥ 1 |
| Most proximal thrombus: Right main pulmonary artery | Thrombus burden | Categorical | Total occlusion; partial occlusion; none |
| Most proximal thrombus: Right upper lobe | Thrombus burden | Categorical | Total occlusion of lobar artery; partial occlusion of lobar artery; segmental arteries; subsegmental arteries; none |
| Most proximal thrombus: Right middle lobe | Thrombus burden | Categorical | Total occlusion of lobar artery; partial occlusion of lobar artery; segmental arteries; subsegmental arteries; none |
| Most proximal thrombus: Right lower lobe | Thrombus burden | Categorical | Total occlusion of lobar artery; partial occlusion of lobar artery; segmental arteries; subsegmental arteries; none |
| Most proximal thrombus: Left main pulmonary artery | Thrombus burden | Categorical | Total occlusion; partial occlusion; none |
| Most proximal thrombus: Left upper lobe | Thrombus burden | Categorical | Total occlusion of lobar artery; partial occlusion of lobar artery; segmental arteries; subsegmental arteries; none |
| Most proximal thrombus: Left lower lobe | Thrombus burden | Categorical | Total occlusion of lobar artery; partial occlusion of lobar artery; segmental arteries; subsegmental arteries; none |

The labelling target comprised 14 predefined items of the institutional structured pulmonary embolism CT reporting template. Allowed values reflect the schema contract used for rule-based extraction and structured-output large language model inference. HU = Hounsfield units, PE = pulmonary embolism, DE-CT = dual-energy CT, ECG = electrocardiogram, RV/LV = right-to-left-ventricle ratio.

## Appendix E2: Technical specifications of large language models

| **Parameter** | **Provider** | **Release Date** | **License** | **Context Length** | **Source** |
| --- | --- | --- | --- | --- | --- |
| Open-weight LLM |  |  |  |  |  |
| Llama 3.3‑70b | Meta | 2024 | Llama Community | 131,072 | Ollama |
| Mistral‑small-22b | Mistral AI | 2024 | Mistral AI Research | 131,072 | Ollama |
| Falcon3‑10b | TII / MegaSys | 2024 | Falcon 3 TII | 32,768 | Ollama |
| Qwen2.5‑72b | Alibaba Group | 2024 | Apache 2.0 | 32,768 | Ollama |
| Qwen2.5‑3b | Alibaba Group | 2024 | Apache 2.0 | 32,768 | Ollama |
| Qwen3‑0.6b | Alibaba Group | 2025 | Apache 2.0 | 40,960 | Ollama |
| Qwen3‑1.7b | Alibaba Group | 2025 | Apache 2.0 | 40,960 | Ollama |
| Qwen3‑4b | Alibaba Group | 2025 | Apache 2.0 | 40,960 | Ollama |
| Qwen3‑8b | Alibaba Group | 2025 | Apache 2.0 | 40,960 | Ollama |
| Qwen3‑14b | Alibaba Group | 2025 | Apache 2.0 | 40,960 | Ollama |
| Qwen3‑30b | Alibaba Group | 2025 | Apache 2.0 | 40,960 | Ollama |
| Qwen3‑32b | Alibaba Group | 2025 | Apache 2.0 | 40,960 | Ollama |
| Deepseek‑R1‑1.5b | DeepSeek | 2025 | MIT | 131,072 | Ollama |
| Deepseek‑R1‑7b | DeepSeek | 2025 | MIT | 131,072 | Ollama |
| Deepseek‑R1‑8b | DeepSeek | 2025 | MIT | 131,072 | Ollama |
| Deepseek‑R1‑14b | DeepSeek | 2025 | MIT | 131,072 | Ollama |
| Deepseek‑R1‑32b | DeepSeek | 2025 | MIT | 131,072 | Ollama |
| Deepseek‑R1‑70b | DeepSeek | 2025 | MIT | 131,072 | Ollama |
| Proprietary LLM |  |  |  |  |  |
| GPT-4o-mini | OpenAI | 2024 | Proprietary | ~ 128,000 | OpenAI |
| GPT-4.1-nano | OpenAI | 2025 | Proprietary | ~ 1,000,000 | OpenAI |
| GPT-4.1-mini | OpenAI | 2025 | Proprietary | ~ 1,000,000 | OpenAI |
| GPT-4.1 | OpenAI | 2025 | Proprietary | ~ 1,000,000 | OpenAI |

Context length refers to the maximum tokens processable in a single prompt-response pair. Sources included model cards, press releases, and official documentation. LLM = large language models.

## Appendix E3: Resource use for manual labelling and LLM inference

| **Parameter** | **Cohort processing Time (h)** | **Time per report (s)** | **Cost per report (cent)** | **CO_2_ per report (g)** |
| --- | --- | --- | --- | --- |
| Radiologists (pooled) | 32.8 | 40.4 (16, 150) | 42.1 (26.5, 57.8) | NA |
| Open-weight LLM |  |  |  |  |
| Falcon3-10b | 9.3 | 11.5 (10.1, 15.2) | 0.04 (0.03, 0.05) | 0.3 (0.3, 0.4) |
| Mistral-small-22b | 17.8 | 22 (19.7, 31.5) | 0.07 (0.06, 0.10) | 0.6 (0.6, 0.9) |
| Llama3.3-70b | 38.7 | 47.7 (42.2, 65) | 0.15 (0.13, 0.20) | 1.3 (1.2, 1.8) |
| Qwen2.5-3b | 4.5 | 5.5 (5.1, 8.3) | 0.02 (0.02, 0.03) | 0.2 (0.1, 0.2) |
| Qwen2.5-72b | 40.7 | 50.1 (44.8, 66.3) | 0.15 (0.14, 0.20) | 1.4 (1.3, 1.9) |
| Qwen3-0.6b | 2.5 | 3.1 (2.6, 6.4) | 0.01 (0.01, 0.02) | 0.1 (0.1, 0.2) |
| Qwen3-1.7b | 2.7 | 3.3 (2.7, 5.2) | 0.01 (0.01, 0.02) | 0.1 (0.1, 0.1) |
| Qwen3-4b | 5.4 | 6.6 (5.8, 8.7) | 0.02 (0.02, 0.03) | 0.2 (0.2, 0.2) |
| Qwen3-8b | 6.7 | 8.3 (6.2, 11.4) | 0.03 (0.02, 0.04) | 0.2 (0.2, 0.3) |
| Qwen3-14b | 9.5 | 11.7 (10.3, 15.9) | 0.04 (0.03, 0.05) | 0.3 (0.3, 0.4) |
| Qwen3-30b | 6.6 | 8.1 (6.6, 15.5) | 0.02 (0.02, 0.05) | 0.2 (0.2, 0.4) |
| Qwen3-32b | 20 | 24.6 (21.4, 32.1) | 0.08 (0.07, 0.10) | 0.7 (0.6, 0.9) |
| Deepseek-R1-1.5b | 3.8 | 4.7 (3.6, 8.7) | 0.01 (0.01, 0.03) | 0.1 (0.1, 0.2) |
| Deepseek-R1-7b | 6.8 | 8.4 (6.5, 14.9) | 0.03 (0.02, 0.05) | 0.2 (0.2, 0.4) |
| Deepseek-R1-8b | 6.7 | 8.2 (6.8, 11.5) | 0.03 (0.02, 0.04) | 0.2 (0.2, 0.3) |
| Deepseek-R1-14b | 11.9 | 14.6 (12.9, 18.7) | 0.05 (0.04, 0.06) | 0.4 (0.4, 0.5) |
| Deepseek-R1-32b | 21.4 | 26.3 (22.3, 34.7) | 0.08 (0.07, 0.11) | 0.7 (0.6, 1.0) |
| Deepseek-R1-70b | 36.8 | 45.3 (38.5, 65.4) | 0.14 (0.12, 0.20) | 1.3 (1.1, 1.8) |
| Proprietary LLM |  |  |  |  |
| GPT 4o-mini | 5.9 | 7.3 (3.1, 33.1) | 0.07 (0.06, 0.09) | NA |
| GPT 4.1-nano | 5.2 | 6.3 (3.5, 21.8) | 0.05 (0.04, 0.05) | NA |
| GPT 4.1-mini | 5.3 | 6.5 (3.4, 40) | 0.18 (0.16, 0.21) | NA |
| GPT 4.1 | 6.8 | 8.3 (2.2, 35.2) | 0.97 (0.88, 1.13) | NA |

Unless otherwise specified, data are means, with ranges shown in parentheses as min-max. Cohort time refers to the total time to process 2923 reports, whereas report time, cost and CO_2_ emissions are averages per individual report. Cost is reported in cent per report (1 cent = €0.01). NA indicates values not available for radiologists or proprietary models. LLM = large language models.

## Appendix E4: Per-reader workload and projected resource use on cohort level

| **Parameter** | **Observed reports (n)** | **Time per cohort (s)** | **Cost per report (€)** | **Projected cohort time (h)** | **Projected cohort cost (€)** |
| --- | --- | --- | --- | --- | --- |
| Radiologist 1 | 365 (12.5) | 37.1 ± 11.4 | 0.42 ± 0.13 | 30.2 ± 9.3 | 1230.6 ± 378.7 |
| Radiologist 2 | 366 (12.5) | 29.2 ± 9.3 | 0.26 ± 0.08 | 23.7 ± 7.6 | 773.5 ± 247.4 |
| Radiologist 3 | 366 (12.5) | 41.6 ± 9.4 | 0.36 ± 0.08 | 33.8 ± 7.6 | 1043.6 ± 235.5 |
| Radiologist 4 | 365 (12.5) | 37.8 ± 10.1 | 0.43 ± 0.11 | 30.7 ± 8.2 | 1252.0 ± 334.2 |
| Radiologist 5 | 365 (12.5) | 47.0 ± 10.2 | 0.58 ± 0.13 | 38.2 ± 8.3 | 1688.7 ± 367.7 |
| Radiologist 6 | 365 (12.5) | 47.6 ± 13.8 | 0.53 ± 0.15 | 38.7 ± 11.2 | 1535.0 ± 443.6 |
| Radiologist 7 | 366 (12.5) | 44.7 ± 13.7 | 0.45 ± 0.14 | 36.3 ± 11.1 | 1310.5 ± 400.5 |
| Radiologist 8 | 365 (12.5) | 37.8 ± 9.3 | 0.34 ± 0.08 | 30.7 ± 7.5 | 1001.8 ± 245.8 |

Rows summarise each of the eight radiologists who contributed manual labels. Observed reports indicate the number of reports read by that radiologist and the percentage of the full evaluation cohort (*n* = 2923). Time per cohort (s) and cost per report (€) are given as mean ± standard deviation over the observed cases. Projected cohort time and projected cohort cost scale each reader's mean per-report values to the full cohort size, assuming a comparable case mix across the cohort. These projections form the basis for the radiologist time and cost ranges reported in Table 1.
